# Enabling Digital Compassion in Digital Health Environments: Modified eDelphi Study to Identify Interprofessional Competencies and Technology Attributes

**DOI:** 10.2196/66547

**Published:** 2025-09-03

**Authors:** David Wiljer, Rebecca Charow, Melody Zhang, Brian Lo, Lydia Sequeira, Nelson Shen, Sanjeev Sockalingam, Peter Rossos, Allison Crawford, Gillian Strudwick

**Affiliations:** 1 University Health Network Toronto, ON Canada; 2 Institute of Health Policy, Management & Evaluation Dalla Lana School of Public Health University of Toronto Toronto, ON Canada; 3 Temerty Faculty of Medicine University of Toronto Toronto, ON Canada; 4 Centre for Addiction and Mental Health Toronto, ON Canada

**Keywords:** compassion, digital compassion, health IT, competency, digital health, health informatics

## Abstract

**Background:**

Health care continues to advance through digital innovation, and technology-enabled processes and interventions are increasingly being introduced to deliver and expand access to care. In this evolving digital health ecosystem, health care professionals (HCPs), learners, and organizations may not be prepared or equipped with the knowledge, skills, and behaviors required to navigate these new digital tools while simultaneously sustaining and integrating compassionate care. Moreover, the tools may not be designed and implemented in a manner that facilitates digital compassion.

**Objective:**

This study aimed to identify (1) core digital compassion competencies for health professionals and (2) digital compassion health IT attributes.

**Methods:**

We conducted this study based on the Delphi method, a consensus-building technique using structured group communication that allows a group of experts to identify competencies and agree on items such as standards and attributes by achieving consensus on a given topic. To encourage enriched discussions, we used a modified eDelphi method, where the first round consisted of a group activity and focus group rather than a questionnaire. Due to COVID-19 pandemic restrictions, the first round was held online. Subsequent rounds consisted of questionnaires administered via email and a web-based survey. Using purposive sampling, participants were recruited from project partners and networks of the research team. A panel of experts across Canada in the fields of compassion, health professional or medical education, and technology was engaged to identify and prioritize professional domains and competency statements, as well as essential attributes for the development and deployment of digital technologies for compassionate care.

**Results:**

A total of 54 experts across Canada were recruited, representing diverse professions including patients or service users, HCPs, administrators, policy makers, health educators, data scientists, health technology designers, and software engineers. Overall, 9 focus groups were conducted and analyzed thematically. Seven domains of digital compassion were identified: (1) digital literacy, (2) patient preference, (3) collaboration and co-design, (4) therapeutic relationship, (5) ethical implications, (6) patient safety, and (7) technology safety. Technology attributes to facilitate digital compassion were also generated. We reached consensus after several subsequent rounds, resulting in 58 digital compassion competency statements and 15 technology attributes.

**Conclusions:**

This study identified a digital compassion framework consisting of competencies for HCPs and attributes for digital technologies that would enhance compassion in virtual care encounters. To promote a cultural shift where technologies are perceived to be not only efficient but also compassionate, practices of co-design, training, and ongoing evaluation and iteration must be prioritized within health care organizations. Future research should explore the adaptability of the professional competencies and technology attributes to specific medical specialties or in patient populations.

## Introduction

### Background

Over the past decade, health care has undergone a monumental transformation. Care delivery has been substantially altered from an experience that was primarily face-to-face and paper based to a world increasingly integrated with digital tools, such as electronic health records (EHRs), telemonitoring, mobile health apps, virtual visit apps, internet-based interventions, and virtual reality “smart” homes [1-4]. Digital technologies have the potential to mitigate current barriers in health care, such as workforce shortages and health care access disparity [5], both of which lead to delayed care and limited resources to meet patients’ health care needs [6,7]. Despite the ability of digital technologies to optimize the efficiency, accessibility, and quality of care [8], this transformation poses new challenges for major stakeholders in the system, including health care organizations, care providers, and patients [9,10].

In this new digital ecosystem, health care professionals (HCPs) often find themselves caring for people in unfamiliar contexts in which patient care has become increasingly tied to and mediated by technology. For example, patients now use technology to take a more active role in the decision-making process and management of their health [11-13]. With >100,000 mobile health apps currently available, many individuals monitor lifestyle habits and health symptoms, seek information on health issues [2], and complete online health risk assessments at their preferred time and place [14]. Furthermore, patients can readily access information such as laboratory test results through a patient portal linked to their EHR [15]. Patients and HCPs are increasingly involved in shared decision-making as patients gain greater confidence in managing their care, ultimately altering the traditional patient–care provider relationship [16]. In turn, as patients become substantially more engaged in using digital technologies as a means to manage their health, HCPs must be “digitally prepared.” Specifically, the rapid transition toward digitalized care requires HCPs to gain proficiency in implementing digital tools in their practice and to deliver compassionate care from a distance [17].

Given that virtual health care services provide access and convenience by overcoming distance, location, or time barriers, the demand becomes higher for HCPs to similarly deliver care at any time and place. However, HCPs report that in comparison to in-person care, delivering virtual care led to greater levels of fatigue [18], most likely due to an extended period of looking at a computer screen. This is in line with the notion of “Zoom fatigue,” a term that surfaced amid the COVID-19 pandemic to describe many people’s experiences of excessive amounts of screen time and information overload, as well as limited physical movements from being in front of a screen continually [19]. Major implications of Zoom fatigue include a greater risk of burnout, anxiety, and physical strain [20,21].

Among HCPs, many reported that along with increased screen time, they also experienced technical difficulties and external distractions and the inability to perform certain clinical procedures on the web [22]. To further exacerbate the problem, HCPs also expressed frustrations and difficulty having to interpret nonverbal cues during teleconsultations [23]. In particular, studies revealed that compared to the traditional, in-person encounter, reading a patient’s body language or facial expressions while delivering remote care was significantly hindered [24-27]. Faced with these challenges, care providers may find it difficult to convey a caring, compassionate demeanor through a digital medium. Furthermore, as stated by Swinglehurst et al [28], for HCPs, balancing the adoption of novel, digital health tools while also delivering compassionate care is a significant challenge. HCPs may lack the adequate learning resources to cope with these shifts in practice and to provide compassionate care in this evolving landscape [28].

In a qualitative study that examined EHR training programs for HCPs, Lynott et al [29] found that only one program underscored the importance of being patient-centered in the digital context by providing formal, structured guidelines for physicians to follow while using a digital health tool with patients. While focusing on the patient experience and compassion has been recognized as a core element of health care delivery, less effort has been made to integrate it into practice. No competency standards exist to guide HCPs in facilitating compassionate care [30-32], particularly in the digital health ecosystem. National associations such as the Canadian Medical Association [33] have stressed the need for current medical curricula to reflect the importance of compassion, given that compassionate care can enhance the patient-provider relationship [32,34-36] and is associated with patient well-being and quality of life [34].

Previous literature has defined compassion on three levels, where being compassionate means (1) recognizing that an individual is suffering, (2) responding to the suffering, and (3) acting to alleviate the suffering [37,38]. Some definitions include the nuances of what *responding to the suffering* entails. For example, in the *Oxford Handbook of Compassion Science*, compassion is (1) awareness of another’s experience of suffering or need, (2) feeling *moved* and (3) recognizing this feeling as a response to the other’s need; (4) making a judgment that the other is suffering; and (5) engaging in a behavior in an attempt to alleviate the suffering [39]. Compassion can be distinguished from empathy or sympathy as compassion is not only one’s ability to be affected by, or to take the perspective of the sufferer [40], but also an action-oriented approach to assuage the pain or suffering of others [41]. In recognizing the advances of technology, and the health care ecosystem becoming ever more digital, it is necessary now more than ever to define and articulate what compassion or “digital compassion” [42-44] means in the digital context.

Although previous studies have suggested that compassion and maintaining therapeutic relationships significantly decrease in digital health care [23,45], a scoping review by Kemp et al [45] revealed that compassion may be augmented through digital interventions. The authors noted that digital health may provide novel ways in which compassionate care may be delivered, such as through telemedicine services where the patient and HCPs are in a comfortable environment that could enhance compassion being expressed and felt on both sides [45]. Emerging literature has begun examining the intersections of compassionate care and digital health technologies [17,44,46,47]. Wiljer et al [46] sought to explore potential implications of digital technologies in the delivery of compassionate care. The authors surfaced considerations around how we can ensure that HCPs are prepared to provide compassion through technology or “digital compassion” [46]. In the conceptual model of digital intersections with compassionate care, Wiljer et al [46] describe six major ways in which technology can be used to create compassion: (1) to create awareness of suffering, (2) to mediate the response to suffering, (3) to be used as an online intervention, (4) to train and coach digital literacy, (5) to deliver care, and (6) to leverage artificial intelligence for patient interactions. However, it is important to note that the notion of digital compassion is continually evolving in tandem with the ever-changing landscape of health care. As it becomes increasingly evident that digitalized care delivery is here to stay, work must be done to systematically explore the concept of digital compassion and to develop professional competencies that can guide health care providers to sustain and enhance compassionate care as “digital health providers.” Gaps that exist in the current medical education literature pertaining to the digital readiness of current and future health care providers must be addressed and expanded to include skills related to digital compassion.

### Objectives

Developing an understanding of digital compassion is essential to redefine what compassion is, and should be, in the context of digital care. The overall objective of the proposed work is to create a culture of digital compassion in the health care system where current and future HCPs will have the appropriate attitude, knowledge, skills, and behaviors required to provide compassionate care in a digital health ecosystem. Specifically, building on previous work in digital compassion [42,43,45,46,48], this study had the following aims:

- To identify essential digital compassion competencies for HCPs
- To identify digital compassion attributes for the development and deployment of health IT

## Methods

### Study Design

This study was grounded in the notion of digital compassion, which forms the theoretical foundation for both the research questions and the design of the study. Specifically, this study draws on the conceptual model proposed by Wiljer et al [46], which explores not only how compassion functions in digital spaces but also how digital tools influence the dynamics of compassionate interactions. The study used a modified eDelphi method, a consensus building technique using structured group communication that allows a group of experts to deal with a complex problem [49]. A common structure of the Delphi method involves the administration of multiple rounds of questionnaires, where each round increases in specificity and iteratively builds upon the results of the previous round. The process is continued until the definition of consensus is met or a predetermined number of rounds are completed. This modified eDelphi method consisted of online focus groups for round 1 and web-based surveys for rounds 2 and 3. Panelists were required to have participated in round 1 to be eligible to participate in rounds 2 and 3. Due to the COVID-19 pandemic self-isolation recommendations, round 1 focus groups were held online through a videoconferencing platform. Table 1 outlines the modified eDelphi process for this study.

**Table 1:** Overview of the digital compassion eDelphi study methodology.

Delphi round	Purpose	Action	Product
Round 1: ideation	Brainstorm domains of digital compassion	List of professional competencies and technological attributes developed through small group activities and large group discussions	Draft list of professional competencies and technological attributes statements and definitions
Round 2: conceptual clarity	Define each domain, assess conceptual clarity, rate importance for each domain, and generate competencies and attributes statements	Statements rated with a median score <5 will be revised for conceptual clarity based on feedback provided	Revised list of professional competencies and technological attribute statements and definitions
Round 3: refinement	Assess competence and attributes statements; refine statements	Statements with an “automatic consensus” score will be included if they have a median score >4	Preliminary list of included professional competency and technological attribute statements
Round 4: consensus	Come to consensus on inclusion and exclusion of competence and attributes statements	Remaining statements achieving a median score >3 and an “acceptable consensus” will be included	Finalized list of professional competency and technological attribute statements

### Study Setting and Recruitment

This study was conducted at the University Health Network (UHN), a large academic health sciences center in Toronto, Ontario, Canada. Recruitment spanned across multiple organizations located across Canada.

The expert panel consisted of individuals who possess deep experiential knowledge (over 5 years of professional or lived experience) in informatics or digital health, medical or health professional education, interprofessional education or practice, compassion research, and patient experience. Eligible individuals include those working in clinical, administrative, informatics, digital health, education, consultancy, research, or policy roles within the health care sector and service users (patients or clients). All individuals must be from Canada and be proficient in English.

Participants were recruited through 2 methods. First, using a purposive sampling strategy, to ensure representation from a variety of professions, perspectives, and work settings. The study team prepared a list of potential participants and organizations from all over Canada and pan-Canadian jurisdictions. This list was in a matrix format where the rows were types of professions and roles in health care and the columns were types of expertise. To capture setting, a color-coding system was used. Organizations (eg, UHN), programs (eg, the Wilson Centre and Patient Experience), and organizational partners of the Associated Medical Services (eg, professional colleges, Canada Health Infoway, Digital Health Canada, and advocacy groups) were approached and asked to disseminate our invitation to their members through their channels (eg, listserve, forums, and newsletters).

Second, a snowball sampling strategy was also used, where participants were asked to share the invitation with those in their network. An informed consent form detailing the study was also appended to the study invitation. The invitation email was re-sent after 3 weeks and after 6 weeks.

Services users (patients and clients) were recruited through patient partner programs and patient advisory groups from the networks of UHN, Associated Medical Services, and Canada Health Infoway.

### Round 1: Ideation and Identification of Initial Delphi Items

The purpose of this round was for panelists to “ideate” the concept of digital compassion as it relates to the conceptual model, health care delivery, professionalism, organizational culture, and technological attributes. We used a novel methodology grounded in narrative inquiry [50], where concept mapping began with an interactive free-writing exercise and real-time content analysis using a digital whiteboard. The format included small breakout sessions for idea generation, followed by a large group discussion to share and prioritize generated ideas. This way, the large group discussion was framed by these narratives, where participants shared their experience and prioritized ideas that were generated from these narrated experiences. At the beginning of each focus group, guidelines for group standards were reviewed, including confidentiality, how to seek support if distressed, and when and how to provide feedback. This was done to create a safe space for participants to speak to and share their experiences.

Round 1 consisted of multiple focus groups following a modified “world café” methodology [51]. In comparison to the traditional approach, where participants would often move from table to table, there was a progressive discussion over a series of focus groups in round 1. Participants did not have to participate in more than one session since each focus group built on the previous session. For example, several focus groups began by reviewing themes that were identified from previous sessions. Furthermore, rather than having large pieces of paper and sticky notes, we used a digital whiteboard, Miro, to capture the discussions. Finally, the focus group script was pilot-tested and then iterated with each round. For instance, the order of questions would be reversed if previous focus groups focused heavily on a certain topic. In each session, a discussion between moderators and panelists was facilitated with broad questions (eg, “What are some technology features that make a digital interaction feel more or less compassionate than an in-person one?”). Focus groups were recorded and transcribed for analysis.

### Round 2: Conceptual Clarity

The list of competencies and attributes from round 1 was circulated to the panelists electronically via a UHN research version of LimeSurvey (LimeSurvey GmbH), a secure web-based survey tool. Panelists assessed the conceptual clarity of each statement on a balanced 6-point Likert scale (1=very unclear to 6=very clear). Participants were given the opportunity to provide suggestions on statement refinement as well as to suggest additional competencies or attributes to include in the list. Consensus was considered to be reached and the statement had received a median score of ≥5, with an IQR <1.5 in round 3. No consensus indicates that a statement had received a median score of ≤4, with an IQR of ≥2.

### Round 3: Refinement

Panelists were provided with a summary of the round 2 results electronically via email, along with a revised list of digital compassion competencies and requirements via REDCap (Research Electronic Data Capture; Vanderbilt University [52,53]), a secure web-based survey tool. In addition, the survey link included a glossary of terms as a downloadable attachment, based on feedback from round 2. Multimedia Appendix 1 [36,54-68] provides the glossary of terms. Panelists rated the importance of each statement on a 7-point Likert scale (1=low priority and 7=high priority) for inclusion. The opportunity for feedback or rationale for each statement rating could be provided through an open-ended textbox. Consensus indicates that a statement had received a median score of ≥5, with an IQR <1.5 in round 3. No consensus indicates that a statement had received a median score of ≤4, with an IQR of ≥2.

### Round 4: Consensus

To reach final consensus, 8 study team members who have research and clinical expertise in competency development, digital health informatics, compassion, medical education, and digital health equity were involved. The members reviewed the list of competency statements and technology attributes, in lieu of the survey panelists. This method was used in consideration of the time commitment that panelists have provided throughout the past 3 eDelphi rounds. In addition, the feedback from the second and third rounds became increasingly circular and, at times, contradictory. Thus, the final statement selection, wordsmithing, and formatting were deferred to the study team and then shared with panelists for final approval.

The final consensus consisted of 4 phases (see Table 2 for a breakdown of round 4). First, the study team reviewed and voted on statements and attributes that had not received overall consensus from the previous round. Using a Microsoft Excel file with 2 tabs, study team members voted asynchronously. One tab had a table with the following columns: statements that had not reached consensus in the previous round, the decision to include (yes or no), and a rationale for the decision. The other tab had a table with the following columns: statements that had reached consensus in the previous round, the domain the statement belonged to, the median score, the IQR, whether the consensus was acceptable or automatic based on the median and IQR, and any panelist feedback on the specific statement. Statements and attributes that did not reach consensus from this asynchronous voting process were then discussed synchronously. To facilitate the discussion, statements were added to a digital whiteboard, Miro, along with a table summarizing the team voting results, any rationale provided for voting “no,” and any panelist feedback from round 3. Two team meetings were devoted to this synchronous voting process, after which it was decided that it was not an efficient use of time.

Second, to further distinguish concepts based on competency level, statements within the 3 themes were arranged in the hierarchical level of taxonomy proposed by Bloom (knowledge, comprehension, application, analysis, synthesis, and evaluation) [69] on a digital whiteboard. Arranging the statements based on learning stages (eg, early stage: knowledge vs late stage: evaluation) enabled the study team members to visualize the learning process captured by the competency statements. Study team members assessed the diversity of statements across the 6 taxonomy levels proposed by Bloom [69]. Repetition or duplication of statements were also examined. A digital whiteboard was used to allow study team members to examine the statements simultaneously. Each member was able to edit, add, or move statements around in real time. In the penultimate step, team members reviewed the final statements and attributes in the context of a care process or clinical encounter on Miro. Finally, the final list of competency statements and technology attributes were shared with panelists from the previous 3 rounds for feedback.

**Table 2:** Round 4 consensus process for reviewing competencies and technology attributes.

Round 4 consensus	Action
Phase 1: review and consensus	Reviewing competencies and technology attributes on a Microsoft Excel file
Phase 2: review and consensus	Reviewing competencies and technology attributes mapped out in Bloom’s taxonomy hierarchical order on Miro
Phase 3: review and consensus	Reviewing competencies and technology attributes mapped out across a clinical encounter on Miro
Phase 4: panelist review	Expert panelists reviewed the final list of competencies and technology attributes

### Data Analysis

#### Overview

Data analysis was conducted in each round of the study. Findings and the format were shared with panelists before progressing on to the next round. By doing so, we engaged in a member-checking process [70] and established an audit trail [71] to enhance validity of the data.

#### Round 1

In the first round, the digital whiteboard used to collect keywords from the narrative stories was shared after the activity. The panelists provided feedback and comments on the findings of the rapid content analysis. This ensured that the appropriate information was captured during the round 1 focus groups.

A thematic analysis was conducted using an iterative, inductive method of constant comparative analysis. The initial coding of the focus group discussions was conducted by 3 independent reviewers (RC, Tharshini Jeyakumar, and Sarah Younus) who had expertise in digital education and health informatics, health services research, patient engagement, and public health. Themes were generated and clustered by 4 reviewers (MZ, Inaara Karsan, Sarmini Balakumar, and RC) independently. These reviewers have foundational backgrounds in mental health, eHealth, health informatics, as well as health services and patient engagement research. After reviewing separately and using memos to keep track of the process, the reviewers convened for group discussions.

The development of competency statements and technology attributes was informed by generated themes. These statements were inputted into LimeSurvey as a survey and forwarded to expert panel members in round 2. Any feedback or comments from panelists were used in the revision process.

#### Round 2 and Round 3

For the web-based survey engagement activities, descriptive statistical analysis (ie, median, IQR, mode, and frequency) was used to determine whether a statement required revision (round 2) or inclusion (round 3), where a lower IQR score indicated greater degrees of consensus. An IQR <1 indicated high consensus (or “automatic consensus”). Statements rated with an IQR of 1.5 and a majority agreement (%) with the median were considered “acceptable consensus.” The feedback from the open-ended questions was used to revise statements or to inform the decision to retain or exclude statements. Consensus in this study was defined as >70% of panel member responses falling within 2 points on the Likert scale [49].

#### Final Round

In the final round, all study team members ranked statements by relevance and importance through an asynchronous review using Microsoft Excel before moving on to a synchronous discussion using Miro board and taxonomy proposed by Bloom. Afterward, the presentation and format of the statements was discussed and reviewed to ensure readability, understandability, and digestibility.

### Ethical Considerations

This study was approved by the UHN Research Ethics Board (CAPCR ID: 19-5832). Several ethical considerations were taken in the development of this study. First, participation in this study required written consent, where all potential expert panel members were sent a consent form along with the recruitment email. The consent form detailed the study activities, the voluntary nature, and confidentiality when participating as well as the benefits and risks of participating in this study. If a potential expert panel member decided to participate in the study, they were free to withdraw at any time without penalty and could choose not to answer a question or rate a competency statement if they did not feel comfortable doing so. In addition, they had the opportunity to notify the principal investigator if they wished to have their data removed from the study.

Information gathered from the in-person activity and web-based surveys were confidential and deidentified. The in-person activities were audio-recorded and transcribed. The transcripts were deidentified before data analysis, and the audio recordings were destroyed after transcription and verification. Each participant who provided consent was assigned a study ID number so that their anonymity can be retained throughout the study. The participant’s name and corresponding study ID number were saved only in the master log. Storage, transportation, and destruction of the study-related documents adhered to UHN Research Ethics Board policy, where all electronic files were stored within a UHN network drive, accessible to only UHN study team members, and destroyed 10 years after completion of the study. Access to raw data was only made available to the UHN research team. External members of the research team who supported the development of this study did not have access to the raw data. Only aggregated data were reported externally, and these files were password protected to restrict access to the external members of the research team. Participants were not compensated for this study.

## Results

### Participant Demographics

Of the 65 individuals who had consented to the study, some did not respond to scheduling requests for the focus group interviews (n=7, 11%), some did not meet the inclusion criteria to be a part of the expert panel (eg, <5 years of professional or lived experience; n=1, 1%), or some were unable to participate due to external circumstances (eg, family emergency; n=3, 5%). In total, 54 panelists participated in round 1, followed by 33 (61%) panelists participating in round 2 and 24 (44%) panelists participating in round 3, and the majority of participants acknowledged the receipt of the final version of the statements, with 2 panelists providing detailed feedback. Diverse professions were represented in each round (see Table 3 for a breakdown of participant demographics for each eDelphi round). For gender, panelists identified themselves as cisgender female and male, while no one identified as transgender male, transgender female, or nonbinary. Panelists were given the option to self-describe if they chose nonbinary. Nonbinary includes, but is not limited to, genderqueer, gender-nonconforming, gender-neutral, agender, and gender-fluid [72]. Finally, no one chose “prefer not to respond.”

**Table 3:** Participant demographics from eDelphi rounds 1 to 3.

Characteristics		Values, n (%)		
		Round 1 (n=54)	Round 2 (n=33)	Round 3 (n=24)
**Gender**				
	Cisgender female	41 (76)	24 (73)	16 (67)
	Cisgender male	13 (24)	9 (27)	8 (33)
	Transgender female	0 (0)	0 (0)	0 (0)
	Transgender male	0 (0)	0 (0)	0 (0)
	Nonbinary	0 (0)	0 (0)	0 (0)
	Prefer not to respond	0 (0)	0 (0)	0 (0)
**Age (years)**				
	18-34	5 (9)	2 (6)	0 (0)
	35-44	11 (20)	6 (18)	4 (17)
	45-54	17 (31)	16 (48)	9 (38)
	55-64	12 (22)	2 (6)	5 (21)
	≥65	4 (7)	2 (6)	2 (8)
	Not specified	5 (9)	5 (15)	4 (17)
**Province**				
	Alberta	4 (7)	2 (6)	2 (8)
	British Columbia	5 (9)	3 (9)	2 (8)
	Manitoba	1 (2)	1 (3)	1 (4)
	Nova Scotia	2 (4)	2 (6)	1 (4)
	Ontario	36 (67)	21 (64)	16 (67)
	Quebec	3 (6)	2 (6)	2 (8)
	Saskatchewan	2 (4)	1 (3)	0 (0)
	Outside of Canada	1 (2)	1 (3)	0 (0)
**Location**				
	Rural	0 (0)	0 (0)	0 (0)
	Small urban	6 (11)	5 (15)	5 (21)
	Large urban	31 (57)	19 (58)	13 (54)
	Not specified	0 (0)	9 (27)	6 (25)
**Education**				
	Bachelor’s	3 (6)	2 (6)	1 (4)
	Master’s	20 (37)	12 (36)	9 (38)
	Applied or professional degree (eg, MD)	2 (4)	1 (3)	3 (13)
	Doctorate degree (eg, PhD)	25 (46)	14 (42)	10 (42)
	Joint MD-PhD degree	4 (7)	4 (12)	1 (4)
**Profession**				
	Health care professional	41 (76)	29 (88)	18 (75)
	Care provider	9 (17)	2 (6)	1 (4)
	Educator-researcher	9 (17)	8 (24)	5 (21)
	Provider-educator or researcher	7 (13)	8 (24)	6 (25)
	Administrator	16 (30)	9 (27)	6 (25)
	Policy maker	1 (2)	0 (0)	0 (0)
	Patient partner	4 (7)	2 (6)	2 (8)
	Informatics or digital health professional	9 (17)	4 (12)	4 (17)

### Round 1

Themes were constructed from the focus group data and went through multiple revisions. The process was 3-fold. First, four domains emerged: (1) digital environment (descriptions and factors related to setting a compassionate response); (2) processes (organizational process, policies, and procedures that would then allow health care providers to deliver compassionate care); (3) professional competencies (digital compassion competencies for health professionals); and (4) technology attributes (digital compassion standards or attributes for the design and implementation of health IT). Within the digital environment domain, clinical interaction, connection, enabling compassionate care, time, and a sense of trust in the system emerged as subthemes. Organizational processes, policies, or procedures that would allow HCPs to deliver compassionate care included choosing the appropriate modality for communication with patients, embedding digital tool use into existing workflows, providing support, and being mindful of whether compassion was considered when selecting digital tools or systems. The professional competencies domain comprised attitudes, knowledge, and skills that HCPs should demonstrate or have when providing digitally compassionate care. As for technology attributes, standards for the design of technology should encompass accessibility, ease of use, layout and seamless interaction, interoperability, security, understandability, and user-centered co-design. Second, similar concepts that emerged across the 4 domains were then consolidated and analyzed. From this, three themes emerged: (1) digital readiness and literacy, (2) fostering connection and digital therapeutic alliance, and (3) patient empowerment and shared decision-making. Theme 1, digital readiness and literacy, encompassed key factors for system readiness, individual readiness, and technology readiness. A quote that best exemplifies this theme is “[H]aving a digital-ready health care system...it would be having a system that’s ready and having professionals who are digitally literate to engage throughout the entire system” (ID27, session 2)*.* Theme 2, fostering connection and digital therapeutic alliance, included important considerations for relationship building, including professionalism and trust, adaptability, as well as awareness of the self and others. A quote that best exemplifies this theme is “Maintaining the therapeutic alliance so that ‘technology’ [does not take] over from the patient” (ID9, Session 1). Finally, theme 3, patient empowerment and shared decision-making, encompassed key considerations for supporting patient engagement since “digital health is a team sport” (ID13, session 2)*.* This included acknowledgment and validation of patient experiences and patient preferences. A quote that best exemplifies this theme is “When the boundaries come down or the hierarchy is leveled, then I think the level of trust that I can put in someone is far greater” (ID28, session 4). Third, statements were iteratively developed for each theme in terms of what care providers, organizations, and technology should do. Statements for what care providers should do were thus grouped into three domains: (1) digital readiness (how to design, adopt, and evaluate technology with compassion in mind); (2) patient engagement (how to engage and collaborate with patients to adopt and feel comfortable using digital health technologies); and (3) relationship building (how to build trust and foster a therapeutic alliance with patients when using digital health technologies). Statements for what technology should do was separated into a stand-alone technology attributes section. A total of 47 competency statements fell under the 3 domains. Of these, 19 (40%) were in digital readiness, 8 (17%) pertained to patient engagement, and 20 (43%) were for relationship building. A total of 16 technology attributes were also developed. Multimedia Appendix 2 provides a list of statements presented to the panelists in the round 2 survey.

### Round 2

A total of 66 statements were included in round 2, with 50 (76%) competency statements and 16 (24%) technology attributes. The average rating for the clarity of the 50 competency statements was “5” or “Clear” across the 3 domains (digital readiness, patient engagement, and relationship building). Specifically, the digital readiness domain was rated 4.75, patient engagement was rated 5.26, and relationship building was rated 5.36. Given that the digital readiness domain was given the lowest ratings, we incorporated participant feedback to refine the statements for round 3. The technology attributes section, which had 16 statements, was given an average rating of 5.46. Multimedia Appendix 3 provides a detailed average clarity rating per statement in the round 2 survey.

### Round 3

Following panelist feedback in round 2, a total of 79 statements were included in round 3, with 60 (76%) competency statements and 19 (24%) technology attributes. The total number of statements was increased due to panelist feedback on several statements being too long or the need for them to be simplified. In this round, panelists were asked to rate the priority of each statement, with 1=low priority to 7=high priority. Overall, statements were given a rating of 6 or high priority across the 3 domains (digital readiness, patient engagement, and relationship building). Specifically, all 3 domains were given an overall rating of 6. In this round, 20 competency statements (6 in relationship building, 1 in patient engagement, and 13 in digital readiness) and 11 technology attributes reached acceptable consensus among the panelists. The remaining 40 competency statements (11 in digital readiness, 12 in patient engagement, and 17 in relationship building) and 8 technology attributes did not receive consensus and were retained for review in the next round. Multimedia Appendix 4 provides the consensus, average priority rating, and consolidated feedback from panelists per statement in the round 3 survey.

### Final Round

#### Review of Nonconsensus Statements From Panelists

The study team members were provided with statements that did or did not reach consensus among the panelists in round 3. The study team members were requested to review statements that had not reached consensus. Statements that the study team members did not agree to retain were removed from the list.

#### Review Statements by Diversity of Taxonomy Proposed by Bloom

Following a review among study team members, there was agreement to remove 7 competency statements due to similarity or repetition with other statements. Through an iterative process over 4 rounds of feedback, a competency framework was established consisting of seven domains, with 58 professional competencies and 15 technology attributes mapped across the domains: (1) digital literacy, (2) ethical implications, (3) collaboration and co-design, (4) patient preferences, (5) therapeutic relationship, (6) patient safety, and (7) technology safety (Table 4). Multimedia Appendix 5 provides the comprehensive list of competencies and attributes.

**Table 4:** List of digital compassion domains, professional competencies, and technology attributes.

Domain	Definition	Professional competencies (n=58), n (%)	Technology attributes (n=15), n (%)
Digital literacy	Navigating, engaging patients, and evaluating the technology	12 (21)	3 (20)
Ethical implications	Considering ethical impact and health equity gaps when using technology	4 (7)	1 (7)
Collaboration and co-design	Advocating for patient preferences in technology design	10 (17)	5 (33)
Patient preferences	Acknowledging patient preferences	11 (19)	1 (7)
Therapeutic relationship	Assessing etiquette when engaging with patients virtually	14 (24)	0 (0)
Patient safety	Ensuring patient’s emotional and physical well-being	3 (5)	2 (13)
Technology safety	Ensuring the safety and confidentiality of a technology	4 (7)	3 (20)

#### Review Format or Review of Contextualizing Statements Across the Care Process or Clinical Encounter

In the final review process, competency statements and technology attributes were conceptualized and mapped across different contexts, *before*, *during*, and *after* a care process or clinical encounter (Table 5), based on panelist feedback and study team discussion. This allowed the study team to visually see where the statements and attributes would fit during a real encounter between an HCP and a patient. Study team members provided feedback on format and clarity. In the final step, themes were broken down further to subthemes and new subcategories *before*, *during*, and *after* a clinical encounter. Subsequently, panelists were engaged and asked to provide final comments and feedback.

**Table 5:** Example of competency statements across a clinical encounter.

Professional competencies				Technology attributes
Before the clinical encounter, a care provider should...	During the clinical encounter, a care provider should...	After the clinical encounter, a care provider should...	Technology should...	

Reflect on one’s own abilities and limitations with technology in clinical settings and recognize when to seek help (digital readiness and technology implementation)	Reflect on how the presence of technology changes an interaction with patients and families and may potentially impact focus on the patient (relationship building and professionalism)	Identify existing evaluation toolkits or checklists to be able to evaluate the technology. (digital readiness and technology evaluation)	Be designed as a seamless experience so that the focus remains on the care and what the health care professional is providing to patients, not the use of technology	
Understand the technology, including its role in providing care to the patient, and how it is integrated within the clinical workflow (relationship building and trust)	Facilitate user engagement by explaining the technology to the patient	Collect data about user experience and engagement with digital tools (digital readiness and technology evaluation)	Be compatible, aligned, or interoperable with other tools for a seamless digital experience for users (patients and care providers)	
Establish communication response times for email and text-based messaging, so all stakeholders can expect when messages are addressed and responded to (relationship building and continuity of care)	Plan follow-up communication with patients who will be receiving laboratory or imaging results through digital health tools, such as patient portals (relationship building and continuity of care)	Assess the follow-up communication plan and whether patients felt like they could ask their care provider questions to understand their results and any required actions or follow-up	Provides personalized prompts, feedback, or education materials and considers users’ functional, cognitive and emotional needs (eg, push notifications to facilitate proactive follow-up or messaging)	

## Discussion

### Principal Findings

In the movement toward digitally compassionate health care, this study identified digital compassion competencies for HCPs and a set of attributes for the development and deployment of health IT. In the formation of our competency framework, an interprofessional expert panel was selected with the goal of representing a wide range of health care and informatics professions. Over iterative rounds of brainstorming, discussion, and feedback, a digital compassion competency framework was established consisting of 7 domains. These domains were mapped onto the conceptual model by Wiljer et al [46] of digital intersections with compassionate care, to align relevant professional competencies and technology attributes to each potential intersection of digital technology and compassionate care. Our framework acknowledges the different potential use cases and designs of technology to facilitate compassionate care (Table 6), highlighting the importance of personalized responses in compassionate care; that is, technology should facilitate case-specific responses, connecting with people based on their characteristics and needs. Moving forward, there may be potential for artificial intelligence to support personal predictive approaches to best meet end-user needs. As described by Wiljer et al [46], the intersection of artificial intelligence and compassion should be further explored to understand how algorithms can identify user emotions and behavior and develop appropriate responses to them.

**Table 6:** Conceptual model and digital compassion framework.

Conceptual model [46]	Competency statement or technology attribute
Awareness of suffering (raising awareness of another’s suffering or need)	Recognize the digital space as an opportunity to understand and address user needs through open discussions and collaboration (patient engagement and shared decision-making)
Mediated response (recognizing suffering and making a judgment that a response is needed)	Identify opportunities where digital health technologies can be used to alleviate suffering and improve patient-oriented outcomes (digital readiness and technology design)
Online intervention (using digital tools to enact compassion)	Evaluate how digital health tools function to enable users (both patients and care providers) to express empathy and emotion with one another (digital readiness and technology design)
Training and coaching (support compassionate approaches to care through online training)	Integrate patient generated data into health assessments, patient history notes, or care planning (patient engagement and patient experience)
Compassion-oriented technologies (digital tools that are designed to convey compassion for end users)	Have communication features for patients to send urgent support requests (during moments of high anxiety or concern) in a safe and secure “live chat” (technology attribute)
Personalized response (artificial emotional intelligence; using algorithms and affective computing to identify relevant emotions; recognize, express, and model emotions; and develop responses)	Provides personalized prompts, feedback, or education materials and considers users’ functional, cognitive, and emotional needs (eg, push notifications to facilitate proactive follow-up or messaging; technology attribute)

The developed competencies and technology attributes reflect the importance of a reciprocally compassionate relationship between the patient and the HCP that considers elements of trust, collaboration, ethical implications, and safety. Digital technologies have permeated many aspects of health care, and to meet the learning needs of HCPs, digital literacy competencies have been widely developed. Yet, these competencies do not give emphasis to compassionate care delivery, with typically a single section on patient-centered applications and education [73,74]. In a scoping review [43] that explored 28 digital health competency frameworks for HCPs, domains within these frameworks highlighted technical competencies, with none of the frameworks focusing on developing or maintaining the digital therapeutic relationship. Furthermore, a large proportion of competency frameworks are developed specifically for nursing and, therefore, may not be generalizable across different health professions. In the compassion literature, while studies have not focused on compassion in digital care, emerging work has examined compassion in health care contexts, such as in pediatric oncology [75]. Conflicting priorities between patients and HCPs are prevalent [75-77]. For instance, Sinclair et al [75] found a mismatch between what patients and families perceived to be important (eg, responding to patient’s suffering with genuine care) and what HCPs perceived to be important (eg, providing care to address medical needs). These conflicts exist in virtual care as well, as reflected in the stories shared by the panelists in our study, where the HCPs prioritized the digital technology (completing a form on the EHR) over genuine acknowledgment of the patient’s suffering.

As such, while great effort has been put on the digital readiness domain (eg, professionalism and digital literacy) [78], elements that contribute to a trusting therapeutic relationship have not been considered sufficiently, such as the ethical implications of access and usability in virtual care, the importance of collaboration and co-design to consider patient preferences, and patient and technology safety when implementing digital tools. Co-design practices often do not have compassion at the center. To enhance engagement and uptake among the patient population, our technology attributes highlight important practices to integrate into the design process to ensure that human-centered design is at the forefront.

The sudden shift to virtual care during the COVID-19 pandemic has exposed unpreparedness to use technology on both sides of the therapeutic relationship [79]. Trust between patients and HCPs remains a common topic in the health system [80,81] as perceived trust can facilitate or disrupt the therapeutic relationship. As we integrate digital tools into care, additional barriers can be created (eg, digital screens in virtual care can increase perceived distance between both parties). Recent work by Shen et al [82] has begun to address that by suggesting the importance of trust-building in virtual clinical encounters through transparent patient consent processes. Thus, it is clear that in the increasingly digitalized health care landscape, it is not sufficient for HCPs to be merely digitally literate. Moving forward, HCPs must have both digital literacy and compassion competencies or “digital compassion” to foster trust and, ultimately, empower patients to engage in the advocacy of their health.

The proposed professional competencies and technology attributes serve as a framework for a cultural shift in the health care ecosystem and to create infrastructure that would support compassionate practices. For patient engagement in health care to reach its full potential, we must emphasize compassion as key as we holistically address issues in virtual care and digital tool usability.

### Limitations and Future Directions

There are several limitations that may affect the generalizability of our findings. First, findings from the round 1 focus groups were based upon what participants shared explicitly and were not observational. Second, there was a limited incorporation of patients and caregivers. Although this study primarily focused on the individual patient, further study focusing on the perspectives of family and caregivers and examining as their role in digitally compassionate care through a digital health equity lens is warranted. This would be of relevance to patients with reduced digital capacity, cognitive ability, or health literacy. In considering the inclusion of patients and caregivers, future directions of this research should also focus on working with specific types of HCPs and clinical sites (eg, cancer care and mental health care). Another limitation is that the definition of digital compassion continues to evolve in consideration of the continuously changing landscape of health care. On the basis of several factors, including inconsistent feedback between rounds, the research team did consensus building directly in round 4, and those decisions were then reviewed by the participant panel. While not ideal, this decision was made to reduce participant burden and to avoid nonproductive iterations of statements. Research is needed to understand how digital compassion may align or distinguish itself from patient-centered care or digital health equity. Further investigations ensue, including who defines when compassion has been provided or experienced and how digital compassion can be measured.

In the next phase of this work, the study team is aiming to create knowledge translation products for major stakeholders, including HCPs, patients and their family and caregivers, and organizational leaders. Our hope is to continue defining the concept of digital compassion and to translate digital compassion competencies beyond the care provider level as described in this paper.

### Conclusions

This eDelphi study identified a framework of professional competencies and technology attributes that have the potential to enhance compassion in virtual care encounters. While the implementation of digital tools and the proficiency in using them to enhance care across all traditional indicators of quality care (accessibility, availability, efficiency, and effectiveness) have had optimistic implications for health care, it is critical that patient experience and fostering trust remain at the center of care delivery. Change requires a cultural shift where all relevant stakeholders are empowered to use technology to promote compassionate care. First, an infrastructure needs to be created to initiate, support, and guide compassion as a core part of the strategic development of virtual care. Second, key stakeholders, including HCPs, patients, families, and external stakeholders who will access health care services via proposed technologies should be engaged in a co-design process. Practices that support sustainability of digital compassion, such as ongoing training, clinical supervision, and resource allocation, should be prioritized. Identifying appropriate evaluation strategies and incorporating patient, family, and community members’ participation and feedback are also necessary. While our competencies and technology attributes serve as the first pieces of the puzzle, future research must be conducted to continue the drive toward a digitally compassionate health care landscape.

## Data Availability

The datasets generated during or analyzed during this study are available from the corresponding author on reasonable request.

## Appendix

Multimedia Appendix 1 Glossary of terms that accompanied eDelphi survey rounds.

Multimedia Appendix 2 List of digital compassion, professional competency, and technology attribute statements for round 2 survey.

Multimedia Appendix 3 Clarity Ratings for Statements in Round 2 Survey.

Multimedia Appendix 4 Priority Ratings, Consensus on Statement Inclusion, and Panelist Feedback from Round 3 Survey.

Multimedia Appendix 5 Final list of digital compassion, professional competency, and technology attribute statements.

## Abbreviations

EHR: electronic health record
HCP: health care professional
UHN: University Health Network
