# Supplementary material for: Enabling Digital Compassion in Digital Health Environments: Modified eDelphi Study to Identify Interprofessional Competencies and Technology Attributes
Source: J Med Internet Res. 2025 Sep 3;27:e66547. doi: 10.2196/66547 (PMC12444226; doi:10.2196/66547)
Supplement: Multimedia Appendix 1 [file jmir_v27i1e66547_app1.docx]

**Appendix 1: Glossary of Terms**

| Active Listening | Listening in an engaged and attentive way while the other party speaks. |
| --- | --- |
| Artificial Intelligence | “A machine with intelligent behavior such as perception, reasoning, learning, or communication and the ability to perform human task [54].” |
| Compassionate care delivery | Delivering healthcare with respect to the patient and their family or caregiver as a result of an attitude, emotion or state where the health care professional is able to “feel for” the patient, relating to  the patient’s experience in some way, recognizing the level of suffering the patient is facing, and resultantly feeling  driven to help. (Adapted from [36]) |
| Communication mediums – Asynchronous vs. Synchronous | Asynchronous - A mode of communication that does not require both parties to be present at the same time  Synchronous - A mode of communication where both parties are present at the same time |
| Digital Fatigue | Physical discomfort experienced after prolonged exposure to a digital screen (e.g., computer, mobile phone, tablet). |
| Digital Health | “Digital health refers to the use of information and communications technologies in medicine and other health professions to manage illnesses and health risks and to promote wellness. Digital health has a broad scope and includes the use of wearable devices, mobile health, telehealth, health information technology, and telemedicine [55].” |
| Digital Literacy (or e-Health Literacy) | The use of emerging information communication technology to improve or enable health or healthcare.  (Adapted from [56]) |
| Digital Therapeutic Alliance | A measure of the quality of the relationship between a patient and provider when using telehealth or digital health interventions. This is associated with effectiveness of digital health interventions and successful care delivery outcomes.  (Adapted from [57]) |
| Electronic Health Records | “a computerized record of a person’s health and/or medical history. This record may contain a person’s full health and medical record, or can be used for certain records, such as lab results, in conjunction with a more traditional paper-based patient chart [58].” |
| Health Equity Gaps | ““[S]ystematic differences in the opportunities groups have to achieve optimal health, leading to unfair and avoidable differences in health outcomes [59].” |
| Health Informatics | “Health informatics is the interprofessional field that studies and pursues the effective uses of biomedical data, information, and knowledge for scientific inquiry, problem-solving, decision making, motivated by efforts to improve human health [60].” |
| Health Information Technology | “Health information technology (HIT) is the hardware, software, and systems that comprise the input, transmission, use, extraction, and analysis of information in the healthcare sector [61].” |
| Human Centered Design | Placing important stakeholders at the center of the design, innovation, and implementation process (Adapted from [62]) |
| Internet-Based Interventions | “For treatments that are mainly delivered via the Internet with at least some therapeutic tasks delegated to the computer [63].” |
| Patient-Generated Data | “Health-related information gathered from patients to help address a health concern, such as self-reported health and treatment histories, physical activity trackers (e.g., FitBit) (HealthIT, Office of the National Coordinator for Health Information Technology; Shapiro et al. 2012). There are two types of generated data: active and passive. Active generated data requires a participant to input details (e.g., mood-tracking), whereas passive generated data does not require participants to input data manually (e.g., sensor-based, remote monitoring) [64].” |
| Patient Portals | “Patient portals give patients safe and secure online access to their personal health information and the ability to manage aspects of their own health care. They also can help patients and clinical teams interact in a more meaningful way to create better patient outcomes [65].” |
| Smart Homes | “Smart home system leverages the Internet of Thing to integrate household devices into a cohesive network, allowing for enhanced control, automation, and efficiency [66].” |
| Structural Inequities | “[P]ersonal, interpersonal, institutional, and systemic drivers—such as, racism, sexism, classism, able-ism, xenophobia, and homophobia—that make those identities salient to the fair distribution of health opportunities and outcomes. Policies that foster inequities at all levels (from organization to community to county, state, and nation) are critical drivers of structural inequities [67].” |
| Tele-Consultation | “Teleconsultation is defined as synchronous or asynchronous consultation using information and communication technology to omit geographical and functional distance. Its goals are for diagnostics or treatment between two or more geographically separated health providers (for example physicians or nurses) or between health providers and patients [68].” |
