# Supplementary material for: Enabling Digital Compassion in Digital Health Environments: Modified eDelphi Study to Identify Interprofessional Competencies and Technology Attributes
Source: J Med Internet Res. 2025 Sep 3;27:e66547. doi: 10.2196/66547 (PMC12444226; doi:10.2196/66547)
Supplement: Multimedia Appendix 3 [file jmir_v27i1e66547_app3.docx]

**Appendix 3: Clarity Ratings for Statements in Round 2 Survey**

**Round 2 Clarity Ratings (Median, IQR) --33 responses**

| **Area/Topic** | **Competent/proficient** | **Median Rating:**  **1 (Very Unclear) - 6 (Very Clear)** |
| --- | --- | --- |
| **Digital Readiness**    ***Technology Design***  ***4.75 (.075)*** |  | 5 (.05) |
|  | Advocate for co-design practices, particularly patient and family involvement in the digital health design process. | 5 (1.5) |
|  | Discriminate when technology use would be a facilitator or barrier to compassionate care delivery. | 4 (1.5) |
|  | Apply human/user centered design thinking, focusing on features that impact compassionate care delivery. | 5 (2) |
|  | Apply human/user centered design thinking, focusing on features that impact how empathy and emotion can be expressed. | 4.5 (3) |
| ***Technology Implementation***  5 (1) | Advocate for digital-ready health care systems and education opportunities to improve digital literacy and bidirectional communication. | 4 (2.5) |
|  | Reflect on personal comfort level with technology in clinical settings. | 5 (1) |
|  | Identify common health equity gaps, vulnerability, structural inequities of patients and families and digital health tool use. | 5 (2) |
|  | Recall digital health technologies available to their patients in their practice setting. | 4 (2) |
|  | Compare the strengths and weaknesses of digital health tools, including its impact on compassionate care delivery. | 5 (1) |
|  | Examine how technology systems can be adapted in new or changing situations (e.g., digital health tools designed for practice setting can be adapted to another). | 5 (1.5) |
|  | Demonstrate digital literacy by being able to navigate/use the digital health technologies implemented in their practice setting and discuss how it compliments and/or replaces care. | 5 (1) |
|  | Demonstrate adaptability when digital health technologies malfunction or fail by problem solving and conflict management. | 5 (2) |
|  | Devise strategies to notify and follow-up with patients when a technology (synchronous or asynchronous communication) fails/malfunctions. | 6 (1) |
|  | Assess when and which digital health tools to employ, keeping in mind patient and family preference, in comparison to in-person or analog (e.g., telephone) options. | 5 (2) |
|  | Critically appraise or validate data presentation or analysis from digital health tools and how to apply this information. | 4 (3) |
| ***Technology Evaluation***  5 (0) | Appraise what elements you do lose in a digital format compared to the in-person experience and how that can be counteracted. | 5 (2) |
|  | Appraise findings from automated and/or artificial intelligence-enabled tools. | 5 (1.5) |
|  | Apply data collection methods to gather feedback on digital health tools use and patient outcomes for continuous practice improvement. | 5 (1.5) |
| **Patient Engagement**    ***Patient Experience***  5 (1) |  | 6 (1) |
|  | Value and allow for time for patients to share their experience when using technology (synchronous or asynchronous communication). | 5 (2) |
|  | Express understanding, validation, and connection by asking the patient how they are feeling and where they are when sharing and discussing lab or imaging results (i.e., are they in the present, are they reminded of something in the past, or are they thinking about their or the future of their loved ones). | 4 (2.5) |
|  | Discuss patient comfort level with digital health technologies that have been recommended to them as well as accessibility concerns, and follow-up with patients over time. | 5 (1) |
|  | Identify the impact of the social determinants of health (e.g., education, culture, lifestyle, economic stability) on preferences related to interest in, use of and experience with digital health technologies in a respectful, nonjudgmental manner. | 5 (2.5) |
|  | Integrate patient generated data into health assessments, patient history notes, and care planning. | 6 (1) |
| ***Shared Decision-Making***  6 (0) | Acknowledge the digital space as a shared space for collaborative compassion and engagement where clear and compassionate discussions, common understanding, and support can be facilitated. | 6 (2) |
|  | Report patient preferences for follow-up forms of communication and appointments, (I.e., synchronous, asynchronous or in-person). | 6 (1.5) |
|  | Prepare clinic workflows and planning activities wherein patients are able to share their communication preferences and digital health technology use. | 6 (1) |
|  | Explain to patients and families digital health tools that could be used for clinical decision support, care planning and/or self management using plain language principles (clear, concise, understandable). | 6 (1) |
|  | Apply goal-oriented care practices to digital health technology use by discussing patient goals and capabilities to express themselves and/or their experience using digital tools. | 6 (2) |
|  | Arrange collaboration opportunities with other health professionals within the patient's circle of care using technology for knowledge exchange and asynchronous and/or synchronous forms of communication for care planning. | 6 (2) |
| **Relationship Building**    ***Professionalism 6 (1)*** |  | 6 (.05) |
|  | Reflect on personal biases and assumptions regarding factors that can influence health outcomes (e.g., education, culture,  economic and housing stability) and digital health literacy of their patients and families. | 5 (2) |
|  | Reflect on how the presence of technology changes an interaction with patients and families and potentially can take over focus from the patient. | 6 (1) |
|  | Reflect on when digital fatigue occurs, what are the resulting behaviours, and strategies for de-compression and time management that may be effective. | 5 (1.5) |
|  | Prepare for patient appointment/consultation by reading over patient history and results from any patient reported outcome measures prior to consultation. | 6 (1) |
|  | Recall key patient history or results from any patient reported outcome measures during the consultation, reducing the number of times the patient electronic record is viewed during the discussion. | 6 (2) |
|  | Express compassion and respect by addressing patients as a person, not a disease or condition. | 6 (0.5) |
|  | Apply active listening when communicating with patients through a synchronous or asynchronous digital medium, and thus respond to patients and families in a thoughtful and personalized way. | 6 (1) |
|  | Demonstrate compassion and respect when communicating with patients through a synchronous, visual digital medium by using animated tone to help convey emotional expression and speaking clearly by using plain language and at understandable, steady pace. (i.e., an awareness of what is being said to a patient and how it is being said.) | 5 (1.5) |
|  | Demonstrate compassion and respect when communicating with patients through a synchronous, visual digital medium through nonverbal actions such as maintaining eye contact looking directly into the camera and being aware of body language and emotional expression. (i.e., an awareness of how they present and act in a digital space and how it differs than an in-person interaction). | 6 (1) |
|  | Demonstrate compassion when communicating with patients through an asynchronous digital medium by writing to them following plain language principles (clear, concise, understandable), convey empathy and emotional expression with emoticons, and personalizing the message, when possible. | 5.5 (1) |
| ***Trust***  6 (0) | Value and encourage a digital therapeutic alliance with patients and families. | 5. 5 (2) |
|  | Discuss the scope of the technology, its role in providing care to the patient, how it is integrated within the clinical pathway, what are the privacy implications, and how their communications and/or data will be secured. | 6 (1) |
|  | Illustrate relatability and honesty by using humor, sharing personal experience, and articulating any difficulty using the technology. | 6 (1) |
|  | Demonstrate emotional resonance - the ability to read and relate to the suffering that is felt by patient and families. | 6 (1) |
|  | Plan phrases or signals with patients to communicate if a patient's environment is no longer safe or private to engage in a discussion about their health. | 6 (0) |
|  | Assess the context and safety of patient's physical and emotional environment during telephone and virtual consultations. | 6 (1) |
|  | Judge the security and confidentiality of the digital applications and equipment that is being used (e.g., secure Internet connection, personal vs. public computer). | 6 (1) |
| ***Continuity of Care***  6 (0.25) | Identify what support systems and patient education resources exist for digital health literacy and how to refer or guide patients on accessing them. (i.e., connect patients with education and resource to access and navigate health information systems as well as understand their health information within these systems). | 5.5 (1) |
|  | Explain to patients how to access and navigate digital health technologies, including patient portals and other health information systems. | 6 (1) |
|  | Devise strategies to proactively communicate/follow-up with patients who will be receiving lab or imaging results and who can access these results through digital health tools, such as patient portals. | 6 (1) |
|  | Setup communication response times for email & text-based messaging, so all stakeholders can expect when messages are sent and will be received. | 6 (1) |
| **Technology Attributes** |  | 6 (0) |
|  | The tool is co-designed with diverse end users (e.g., different language proficiencies, education levels, health literacy levels, digital literacy levels, physical and mental disabilities, etc.). | 6 (1) |
|  | The tool is designed following end-user design principles where there is consideration for features that (1) create a sense of connection and build trust and (2) can be translated or adapted for people with physical and mental disabilities (e.g., is it hearing someone’s voice? Making eye contact?, etc.). | 5 (2) |
|  | The tool is intuitive and easy to use with features that are accessible to a diverse user population (e.g., different language proficiencies, education levels, health literacy levels, digital literacy levels, physical and mental disabilities, etc.). | 6 (1) |
|  | The tool has a user-friendly interface or layout. | 6 (1) |
|  | The tool has an option to give access or facilitate engagement with family or informal caregivers so that they can be included in a patient’s circle of care. | 6 (1) |
|  | The tool has an option to give access or facilitate engagement with other health care professionals in a patient’s circle of care. | 6 (1) |
|  | Written and visual components of a digital tool follow plain language best practices (i.e., content is designed to be understandable and actionable for individuals with low health literacy). | 6 (1) |
|  | The tool is designed to be a seamless experience so that the focus remains on the care and what the health care professional is providing to patients, not the use of technology. | 6 (1) |
|  | The tool can be scaled and integrated within existing clinical workflows and does not require equipment beyond what is typically available to health care professionals in their practice settings (e.g., computer with internet connection, built in webcam, speakers, and microphone, etc.). | 6 (1) |
|  | The tool is interoperable with other digital health tools for a cohesive digital experience. | 6 (1) |
|  | If results can be accessed by patients, ensure there is a mechanism for patients to be able to easily follow-up with their provider regarding understanding their results (i.e., proactive follow-up, messaging, and/or resource connection). | 6 (1) |
|  | The tool provides personalized prompts, feedback or education materials and considers users’ feelings or emotions by sending notifications and/or suggestions based on user activity. | 6 (1) |
|  | Communication features of a digital tool include features that help users convey empathy and emotions (e.g., video, audio, emojis for text messaging, etc.). | 6 (1) |
|  | The tool has a notification feature for when space or connection is no longer safe or secure. | 6 (1) |
|  | The tool has a notification feature for when connectivity or system failures occur and instructions on who to contact and how to contact them are clear. | 6 (1) |
|  | The tool enables users to run a diagnostic of the system to verify the stability, reliability and confidentiality to provide a safe space for both the patient and providers. | 6 (1) |
