# Supplementary material for: Enabling Digital Compassion in Digital Health Environments: Modified eDelphi Study to Identify Interprofessional Competencies and Technology Attributes
Source: J Med Internet Res. 2025 Sep 3;27:e66547. doi: 10.2196/66547 (PMC12444226; doi:10.2196/66547)
Supplement: Multimedia Appendix 4 [file jmir_v27i1e66547_app4.docx]

**Appendix 4: Consensus, Priority Ratings and Panelist Feedback from Round 3 Survey**

**Round 3 Priority Ratings (Median, IQR) -- 24 responses**

| **Area/Topic** | **Competent/proficient (phrasing reviewed by panelists)** | | **Competency/proficient**  **(phrasing suggested by panelists)** | | **Median (IQR)**  1 to 7  (low to high priority) | | **Consensus**  (yes/no) |
| --- | --- | --- | --- | --- | --- | --- | --- |
| **Digital Readiness**  ***Technology Design***  ***6 (1.5)*** |  |  | | 6 (1) | |  | |
|  | 1. Advocate for co-design practices, particularly patient and family involvement. | 1. Advocate for co-design practices where patients and their families are involved. | | 7 (2) | | no | |
|  | 1. Identify when technology use would be a facilitator or barrier to compassionate care delivery. | 1. Identify when technology use may be a facilitator or barrier to compassionate care delivery. | | 6 (2) | | no | |
|  | 1. Discuss how compassionate care delivery is perceived by end-users (both patients and providers) during the design and development of a digital tool by taking a human-centered design approach. | 1. Identify how compassionate care delivery is perceived by end-users (both patients and providers) during the design and development of a digital tool by taking a human-centered design approach. | | 6 (1.5) | | yes | |
|  | 1. Assess how features of a digital health tool impact how users (both patients and providers) can express empathy and emotion with one another. | 1. Assess how digital health tools function to enable users to express (both patients and providers) empathy and emotion with one another. | | 5 (2) | | no | |
| ***Technology Implementation***  ***6 (1)*** | 1. Identify opportunities to transition into digital systems. | 1. Identify opportunities to transition into digital systems. | | 5 (2) | | no | |
|  | 1. Identify education opportunities for all stakeholders (patients, families, healthcare providers) to improve digital literacy and digital communication methods). | 1. Identify education opportunities for all stakeholders (patients, families, healthcare providers) to improve digital literacy and digital communication methods. | | 6 (2) | | no | |
|  | 1. Reflect on your own personal comfort level with technology in clinical settings and when to seek help. | 1. Reflect on one’s own abilities and limitations with technology in clinical settings and recognize when to seek help. | | 5 (1) | | yes | |
|  | 1. Identify when digital health tools can be used to address an issue you are facing or when making clinical decision. | 1. Identify opportunities where digital health technologies can be used to improve outcomes and/or support clinical decision making. | | 5 (2) | | no | |
|  | 1. Identify common health equity gaps, vulnerability, structural inequities of patients and families and digital health tool use (e.g., systematic drivers of bias, where some patient populations are at more of an advantage/disadvantage than others). For instance, where non-native English speakers do not receive as much benefit as a native English speaker because the information on a digital tool. | 1. Recognize common health equity gaps, vulnerability, structural inequities of patients and families related to digital health technologies (e.g., where some patient populations are at more of an advantage/ disadvantage than others). For instance, where non-native English speakers do not receive as much benefit as a native English speaker because the information on a digital tool. | | 6 (2) | | no | |
|  | 1. Demonstrate awareness of digital health technologies and resources that exist for providers, patients, and their families at your healthcare practice | 1. Demonstrate awareness of digital health technologies and resources that exist for patients and their families at your healthcare practice by providing information when needed. | | 6 (1.5) | | yes | |
|  | 1. Compare the strengths and weaknesses of digital health tools, including its impact on compassionate care delivery. | 1. Compare the strengths and limitations of digital health tools, including the impact on compassionate care delivery. | | 5 (2) | | no | |
|  | 1. Identify opportunities to adapt technologies from one setting to another. | 1. Identify opportunities to adapt technologies from one setting to another. | | 5 (2) | | no | |
|  | 1. Demonstrate digital literacy by being able to navigate and use the digital health technologies implemented in your healthcare practice. | 1. Demonstrate digital literacy by being able to navigate and use the digital health technologies implemented in your healthcare practice. | | 6 (1) | | yes | |
|  | 1. Describe how digital health technologies complement or enable in-person care in your healthcare practice. | 1. Describe how digital health technologies enhance or enable in-person care in your healthcare practice. | | 5 (2) | | no | |
|  | 1. Describe the ethical implications when using digital health technologies to complement or enable in-person care in your healthcare practice. | 1. Describe the ethical implications when using digital health technologies to complement or enable in-person care in your healthcare practice. | | 6 (1) | | yes | |
|  | 1. Demonstrate adaptability by problem solving when digital health technologies malfunction or fail (when the link, connection, or space is no longer secure). | 1. Demonstrate adaptability by problem solving when digital health technologies malfunction or fail (when the technology does not function as expected). | | 6 (2) | | no | |
|  | 1. Devise strategies to manage potential conflict with your patient(s) by notifying and following up with them when a technology (synchronous or asynchronous communication) malfunction or fail. | 1. Devise communication strategies with your patient(s) by notifying and following up with them when a technology (synchronous or asynchronous communication) malfunctions or fail. | | 6 (2) | | no | |
|  | 1. Assess when and which digital health tools to employ, keeping in mind patient and family preference. | 1. Assess when and which digital health tools to employ, keeping in mind patient and family preference. | | 6 (2) | | no | |
|  | 1. Review and make meaning of data from digital health tools. | 1. Review and interpret data from digital health technologies to improve care. | | 6 (1.25) | | yes | |
| ***Technology Evaluation***  ***5.5 (0.75)*** | 1. Identify elements that detract from care in a digital format, and devise solutions for improving experiences for patients and providers. | 1. Identify elements that detract from care in a digital format, and devise solutions for improving experiences for patients and providers. | | 5.5 (1.5) | | yes | |
|  | 1. Identify existing evaluation toolkits or checklists to be able to evaluate the technology. | 1. Identify existing evaluation toolkits or checklists to evaluate the technology. | | 5 (1.5) | | yes | |
|  | 1. Evaluate the relevance, trustworthiness, and ethical use of data from digital clinical decision support tools, such as automated and/or artificial intelligence-enabled tools. | 1. Evaluate the relevance, trustworthiness, and ethical use of data from digital clinical decision support tools, such as automated and/or artificial intelligence-enabled tools. | | 6 (2) | | no | |
|  | 1. Collect data on user feedback from digital tools | 1. Collect data on user feedback from digital tools. | | 5.5 (1.5) | | yes | |
|  | 1. Describe how to utilize user feedback for continuous improvement | 1. Know how to collect and utilize user feedback from digital tools for continuous improvement. | | 5 (2) | | no | |
| **Patient Engagement**  ***Patient Experience***  ***6 (0)*** |  |  | | 6 (0) | |  | |
|  | 1. Acknowledge patients’ experiences with using technology (synchronous or asynchronous communication). | 1. Respect varying experiences that different patients may have with using technology (synchronous or asynchronous communication) and create time for them to share their experiences. | | 6 (2) | | no | |
|  | 1. Create time for patients to share their experiences with using technology (synchronous or asynchronous communication) during a clinical encounter | 1. Acknowledge patient experiences with using technology (synchronous or asynchronous communication) and create time for patients to share their experiences. | | 5.5 (2) | | no | |
|  | 1. Express understanding, validation, and connection by asking the patient about their emotional state n when sharing and discussing their health information, such as lab or imaging results and clinical documentation. | 1. Engages with and acknowledges patient emotional state when sharing and discussing their health information, such as lab or imaging results and clinical documentation. | | 6 (2) | | no | |
|  | 1. Discuss patient comfort level and accessibility concerns with recommended digital health technologies | 1. Discuss patient comfort level and accessibility concerns with recommended digital health technologies | | 6 (2) | | no | |
|  | 1. Consider the ethical implications and impact of the social determinants of health (e.g., education, culture, housing, internet accessibility) on preferences related to using digital health technologies in a respectful, nonjudgmental manner. | 1. Recognize the ethical implications and impact of the social determinants of health (e.g., education, culture, housing, internet accessibility) on preferences related to using digital health technologies in a respectful, nonjudgmental manner. | | 6 (1) | | yes | |
|  | 1. Integrate patient generated data into health assessments, patient history notes, and care planning. | 1. Integrate patient generated data into health assessments, patient history notes, and care planning. | | 6.5 (2.25) | | no | |
| ***Shared Decision-Making***  ***6 (0)*** | 1. Recognize the digital space as a collaborative space where clear and compassionate discussions, common understanding, and support can be facilitated. | 1. Recognize the digital space as a collaborative space where clear and compassionate discussions, common understanding, and support can be facilitated. | | 6 (2.5) | | no | |
|  | 1. Discuss the value of non-digital supports and offline experiences with patients and families and when they would prefer using digital health tools. | 1. Recognize the value of in-person clinical experiences with patients and families and when they would prefer using digital health tools. | | 5 (2) | | no | |
|  | 1. Prepare clinic workflows and planning activities wherein patients are able to use their preferred communication method and digital health technology. | 1. Prepare clinic workflows and planning activities where patients are able to use their preferred communication method and digital health technology. | | 6 (2) | | no | |
|  | 1. Recognize patient preferences for follow-up forms of communication and appointments, (I.e., synchronous, asynchronous or in-person). | 1. Implement patient preferences for follow-up forms of communication and appointments, (I.e., synchronous, asynchronous or in-person). | | 6 (2) | | no | |
|  | 1. Explain to patients and families the digital health tools that could be used for care decision support, care planning and/or self-management using clear, concise, plain language. | 1. Communicate the value of digital health tools that could be used for care decision support, care planning and/or self-management using clear, concise, plain language to patients and families. | | 6 (2) | | no | |
|  | 1. Create goals of treatment with patient and allow patients to identify their comfort with digital tools | 1. Create goals of treatment in partnership with patients and help patients identify their comfort level with digital tools. | | 6 (2) | | no | |
|  | 1. Arrange collaboration opportunities with other health professionals within the patient's circle of care using technology for knowledge exchange and asynchronous and/or synchronous forms of communication for care planning. | 1. Arrange collaboration opportunities with health care team using technology for knowledge exchange and asynchronous and/or synchronous forms of communication for care planning. | | 6 (2) | | no | |
| **Relationship Building**  ***Professionalism***  ***6 (1.5)*** |  |  | | 6 (1) | |  | |
|  | 1. Reflect on personal biases and assumptions regarding factors that can influence health outcomes (e.g., education, culture, economic and housing stability, digital health literacy) of your patients and their families. | 1. Acknowledge on personal biases and assumptions regarding factors that can influence health outcomes (e.g., education, culture, economic and housing stability, digital health literacy) of your patients and their families. | | 6 (2) | | no | |
|  | 1. Reflect on how the presence of technology changes an interaction with patients and families and potentially can take over focus from the patient. | 1. Reflect on how the presence of technology changes an interaction with patients and families and may potentially impact focus on the patient. | | 6 (1) | | yes | |
|  | 1. Reflect on resulting behaviours when digital fatigue occurs and strategies for de-compression (e.g., taking a break from technology). | 1. Reflect on resulting behaviours when digital fatigue occurs and strategies for de-compression (e.g., taking a break from technology). | | 5 (1) | | yes | |
|  | 1. Prepare for patient appointment or consultation by reading over patient history and results from available patient-generated data prior to consultation. | 1. Recognize the importance of reviewing patient generated data in addition to patient history prior to consultation | | 7 (2) | | no | |
|  | 1. Express compassion and respect by being mindful of the number of times the patient electronic record is viewed during the consultation. | 1. Express compassion and respect by being mindful of the number of times the patient electronic record is viewed during the consultation. | | 5 (1) | | yes | |
|  | 1. Express compassion and respect by addressing and referring to a patient as a person, not a disease or condition whether or not that individual is present. | 1. Express compassion and respect by addressing and referring to a patient as a person, not a disease or condition whether or not that individual is present. | | 7 (1) | | yes | |
|  | 1. Apply active listening when communicating with patients through a synchronous or asynchronous digital medium and thus respond to patients and families in a thoughtful and personalized way. | 1. Apply active listening when communicating with patients through a synchronous or asynchronous digital medium and thus respond to patients and families in a thoughtful and personalized way. | | 6.5 (1) | | yes | |
|  | 1. Demonstrate compassion and respect by delivering negative news to patients and families in their preferred form of communication (e.g., telephone, video, email or direct messaging). | 1. Demonstrate compassion and respect by delivering negative news to patients and families in their preferred form of communication (e.g., telephone, video, email or direct messaging). | | 6 (2) | | no | |
|  | 1. Demonstrate compassion and respect when communicating with patients through a synchronous, visual digital medium by conveying emotional expression, using plain language, and speaking at a steady pace) | 1. Demonstrate compassion and respect when communicating with patients through a synchronous, visual digital medium by conveying emotional expression, using plain language, and speaking at a steady pace) | | 6 (2) | | no | |
|  | 1. Demonstrate compassion and respect when communicating with patients through a synchronous, visual digital medium by maintaining eye contact (looking directly into the camera and being aware of body language and emotional expression). | 1. Demonstrate compassion and respect when communicating with patients through a synchronous, visual digital medium by maintaining eye contact (looking directly into the camera and being aware of body language and emotional expression). | | 5.5 (2) | | no | |
|  | 1. Demonstrate compassion when communicating with patients through an asynchronous digital medium by using plain language, conveying empathy and emotions through tone, body language, or emoticons and personalizing the message, when possible. | 1. Demonstrate compassion when communicating with patients through an asynchronous digital medium by using plain language, conveying empathy and emotions through tone, body language, or emoticons and personalizing the message, when possible. | | 5 (2.5) | | no | |
| ***Trust***  ***5.75 (0.75)*** | 1. Demonstrate and encourage a digital therapeutic alliance with patients and families. | 1. Demonstrate and encourage a digital therapeutic relationship with patients and families. | | 5 (2) | | no | |
|  | 1. Describe the technology, its role in providing care to the patient, how it is integrated within the clinical pathway, what are the privacy implications, potential risks, and how their communications and/or data will be secured. | 1. Describe the technology, its role in providing care to the patient, how it is integrated within the clinical pathway, in addition to the privacy implications, potential risks, and how communications and/or data will be secured. | | 6 (2) | | No | |
|  | 1. Demonstrate relatability and honesty by using humility and sharing personal experiences and difficulties using the technology. | 1. Demonstrate relatability and honesty by using humility and sharing personal experiences and difficulties using the technology. | | 5 (2) | | no | |
|  | 1. Demonstrate the ability to read and use verbal/visual cues to acknowledge the suffering that is felt by patient and families. | 1. Demonstrate the ability to interpret and use verbal/visual cues to acknowledge the suffering that is felt by patient and families. | | 6 (2) | | no | |
|  | 1. Plan phrases or signals with patients to communicate if a patient's environment is no longer safe or private to engage in a discussion about their health. | 1. Plan phrases or signals with patients to communicate if a patient's environment is no longer safe or private to engage in a discussion about their health. | | 5.5 (1.25) | | yes | |
|  | 1. Assess the safety of patient's physical and emotional environment during telephone and virtual consultations. | 1. Assess the safety of patient's physical and emotional environment during telephone and virtual consultations. | | 6 (2) | | no | |
|  | 1. Assess the security and confidentiality of digitally stored personal health information. | 1. Assess the security and confidentiality of digitally stored personal health information. | | 6 (1.25) | | yes | |
|  | 1. Assess the security and confidentiality of the digital applications and equipment that are being used (e.g., secure Internet connection, personal vs. public computer). | 1. Confirm the security and confidentiality of the digital applications and equipment that are being used (e.g., secure Internet connection, personal vs. public computer). | | 5.5 (2) | | no | |
| ***Continuity of Care***  ***6 (0.75)*** | 1. Identify what support systems and patient education resources exist for digital health literacy and how to refer or guide patients in accessing them. | 1. Identify what support systems and patient education resources exist for digital health literacy and how to refer or guide patients in accessing them. | | 6 (2) | | no | |
|  | 1. Explain to patients how to access and navigate digital health technologies, including patient portals and other health information systems. | 1. Explain to patients how to access and navigate digital health technologies, including patient portals and other health information systems. | | 5 (2) | | no | |
|  | 1. Plan follow-up communication with patients who will be receiving lab or imaging results through digital health tools, such as patient portals. | 1. Plan follow-up communication with patients who will be receiving lab or imaging results through digital health tools, such as patient portals. | | 6.5 (2) | | no | |
|  | 1. Setup communication response times for email & text-based messaging, so all stakeholders can expect when messages are addressed and responded to. | 1. Setup communication response times for email & text-based messaging, so all stakeholders can expect when messages are addressed and responded to. | | 6 (2) | | no | |

**Digital Compassion Technology Attributes**

| **Area/Topic** | **Statement (phrasing reviewed by panelists)** | **Statement (phrasing suggested by panelists)** | **Median (IQR)** | **Consensus** (yes/no) |
| --- | --- | --- | --- | --- |
| **Technology Attributes** |  |  | 6 (1) |  |
|  | 1. The tool is co-designed following end-user design with features that create a sense of connection and build trust between any end-users (e.g., How can connection be maintained for populations that are visibly impaired?) | 1. The tool is co-designed with features that create a sense of connection and build trust between any end-users (e.g., How can connection be maintained for populations that are visibly impaired?) | 6 (1) | yes |
|  | 1. The tool is easy to use with features that are accessible to a diverse user population (e.g., different language proficiencies, health and digital literacy levels, and disabilities). | 1. The tool is easy to use with features that are accessible to a diverse user population (e.g., different language proficiencies, health and digital literacy levels, and disabilities). | 7 (1) | yes |
|  | 1. The tool is intuitive where it has a user-friendly interface or layout. | 1. The tool has a user-friendly interface and layout, making its use intuitive. | 7 (2) | no |
|  | 1. The tool has an option to give access to or facilitate engagement with family or informal caregivers so that they can be included in a patient’s circle of care. | 1. The tool has an option to give access to or facilitate engagement with family or informal caregivers so that they can be included in a patient’s circle of care. | 6 (2) | no |
|  | 1. The tool has an option to give access to or facilitate collaboration with other health care professionals in a patient’s circle of care. | 1. The tool has an option to give access to or facilitate collaboration with other health care professionals in a patient’s circle of care. | 6 (2) | no |
|  | 1. Written and visual components of a tool follow plain language best practices (i.e., content is designed to be understandable and actionable for individuals with low health literacy). | 1. Written and visual components of a tool follow plain language best practices (i.e., content is designed to be understandable and actionable for individuals with low health literacy). | 6 (2) | no |
|  | 1. The tool is designed to be a seamless experience so that the focus remains on the care and what the health care professional is providing to patients, not the use of technology. | 1. The tool is designed to be a seamless experience so that the focus remains on the care and what the health care professional is providing to patients, not the use of technology. | 7 (2) | no |
|  | 1. The tool can be scaled and integrated within existing clinical workflows and does not require equipment beyond what is typically available to health care professionals in their practice settings (e.g., computer with internet connection, built in webcam, speakers, and microphone). | 1. The tool can be scaled and integrated within existing clinical workflows and does not require equipment beyond what is typically available to health care professionals in their practice settings (e.g., computer with internet connection, built in webcam, speakers, and microphone). | 6 (1) | yes |
|  | 1. The tool is compatible with other tools for a seamless digital experience for users (patients and providers). | 1. The tool is compatible with other tools for a seamless digital experience for users (patients and providers). | 5 (2) | no |
|  | 1. If results can be accessed by patients, ensure patients have a clear plan to follow-up with their provider regarding understanding their results (i.e., proactive follow-up or messaging). | 1. If results can be accessed by patients, ensure patients have a clear plan to ask their provider questions to understand their results (i.e., proactive follow-up or messaging). | 6 (2) | no |
|  | 1. If results can be accessed by patients, education is provided where results are contextualized (e.g., normal lab ranges) and further resources can be accessed. | 1. If results can be accessed by patients, educational information is provided to interpret results (e.g., normal lab ranges) and further resources can be accessed. | 6 (1) | yes |
|  | 1. The tool provides personalized prompts, feedback or education materials and considers users’ feelings or emotions by sending notifications and/or suggestions based on user activity. | 1. The tool provides personalized prompts, feedback or education materials and considers users’ feelings or emotions by sending notifications and/or suggestions based on user activity. | 5 (2) | no |
|  | 1. Communication features of a tool include an option for patients to send urgent support requests (during moments of high anxiety or concern) in a safe and secure ‘Live Chat.’ | 1. Communication features of a tool include an option for patients to send urgent support requests (during moments of high anxiety or concern) in a safe and secure ‘Live Chat.’ | 6 (1) | yes |
|  | 1. Communication features of a tool include features that help users convey empathy and emotions (e.g., video, audio). | 1. Communication features of a tool include features that help users convey empathy and emotions (e.g., video, audio). | 5 (1) | yes |
|  | 1. The tool has a notification feature for when space or connection is no longer safe or secure. | 1. The tool has a notification feature for when space or connection is no longer safe or secure. | 5 (2) | no |
|  | 1. The tool has a notification feature for users (patients and providers) when connectivity or system failures occur. | 1. The tool has a notification feature for users (patients and providers) when connectivity or system failures occur. | 6 (1.25) | yes |
|  | 1. The tool has clear instructions on who to contact and how to contact them if connectivity or system failures occur. | 1. The tool has clear instructions on who to contact and how to contact them if connectivity or system failures occur. | 6 (1.5) | yes |
|  | 1. The tool enables users (patients and providers) to run a diagnostic of the system to verify the stability, reliability and confidentiality. | 1. The tool enables users (patients and providers) to run a diagnostic of the system to verify the stability, reliability and confidentiality. | 5 (1.5) | yes |
|  | 1. Risk assessment plans and strategies are available so that everyone knows what to do if a crisis should arise. | 1. Risk assessment plans and strategies are available so that everyone knows what to do if a crisis should arise (e.g., technology malfunction or patient crisis). | 6 (1) | yes |
