# Supplementary material for: Enabling Digital Compassion in Digital Health Environments: Modified eDelphi Study to Identify Interprofessional Competencies and Technology Attributes
Source: J Med Internet Res. 2025 Sep 3;27:e66547. doi: 10.2196/66547 (PMC12444226; doi:10.2196/66547)
Supplement: Multimedia Appendix 5 [file jmir_v27i1e66547_app5.pdf]

Appendix 5: List of Digital Compassion Professional Competency and Technology Attribute Statements Grouped from the eDelphi study

**PROFESSIONAL COMPETENCIES**			**TECHNOLOGY ATTRIBUTES**
**Provider should…**			**Technology should…**
**Before the clinical encounter**	**During the clinical encounter**	**After the clinical encounter**	
**DIGITAL LITERACY**			
***Demonstrate* digital literacy by being able to navigate and use the digital health technologies implemented in your healthcare practice. (Dig Readiness, Tech Implementation)**	***Describe* how digital health technologies enhance or enable in-person care in your healthcare practice. (Dig Readiness, Tech Implementation)**	***Utilize* user feedback from digital tools for continuous improvement. (Digital Readiness - Technology Evaluation)**	
Reflect on one's own abilities and limitations with technology in clinical settings and recognize when to seek help. (Dig Readiness, Tech Implementation)	*Reflect* on how the presence of technology changes an interaction with patients and families and may potentially impact focus on the patient. (Relationship Building, Professionalism)	*Identify* existing evaluation toolkits or checklists to be able to evaluate the technology. (Dig Readiness, Tech Evaluation)	Be designed as a seamless experience so that the focus remains on the care and what the health care professional is providing to patients, not the use of technology.
Understand the technology, including its role in providing care to the patient, and how it is integrated within the clinical workflow (Relationship Building, Trust)	*Facilitate* user engagement by explaining the intended use of the technology to the patient	*Collect* data about user experience and engagement with digital tools. (Dig Readiness, Tech Evaluation)	Be compatible, aligned or interoperable with other tools for a seamless digital experience for users (patients and providers) to the extent possible.
Identify when technology use may be a facilitator or barrier to compassionate care delivery. (Dig Readiness, Tech Design)	*Review* and interpret data from digital health technologies to improve care (Dig Readiness, Tech Implementation)		
Identify the impact of adopting and adapting technologies from one setting to another. (Dig Readiness, Tech Implementation)			Be scaled and integrated within existing clinical workflows and does not require equipment beyond what is typically available to health care professionals in their practice settings (e.g., computer with internet connection, built in webcam, speakers, and microphone).

<u>Appendix 5: List of Digital Compassion Professional Competency and Technology Attribute Statements Grouped from the eDelphi study</u>

PROFESSIONAL COMPETENCIES			TECHNOLOGY ATTRIBUTES
**Provider should…**			**Technology should…**
**Before the clinical encounter**	**During the clinical encounter**	**After the clinical encounter**	
**ETHICAL IMPLICATIONS**			
Describe **the ethical implications when using digital health technologies to complement or enable in-person care in your healthcare practice. (Dig Readiness, Tech Implementation)**			
Recognize the relevance, and ethical use, sharing and policies around commercialization of data from digital clinical decision support tools to enhance trustworthiness. (Dig Readiness, Tech Implementation)			Be easy to use with features that are accessible to a diverse patient and provider user population (e.g., different language proficiencies, health and digital literacy levels, and disabilities)
Recognize common health equity gaps, structural inequities of patients and families related to digital health technologies (e.g., where some patient populations are at more of an advantage/disadvantage than others). (Dig Readiness, Tech Implementation)			
Recognize the ethical implications and impact of the social determinants of health (e.g., education, culture, housing, internet accessibility) on preferences related to using digital health technologies in a respectful, nonjudgmental manner. (Patient Engagement, Patient Experience)			

PROFESSIONAL COMPETENCIES			TECHNOLOGY ATTRIBUTES
**Provider should…**			**Technology should…**
**Before the clinical encounter**	**During the clinical encounter**	**After the clinical encounter**	
**COLLABORATION AND CO-DESIGN**			
***Arrange* opportunities with their health care team to use technology for collaborative care planning (Patient Engagement, Shared Decision Making)**	***Enable* patients to access and navigate digital health technologies, including patient portals and other health information systems, to the extent possible. (Relationship Building, Continuity of Care)**		
Recommend and guide patients' access to support systems and patient education resources for digital health literacy (Relationship Building, Continuity of Care)	*Demonstrate* the ability to identify appropriate resources or support that exist for patients and their families when needed. (Dig Readiness, Tech Implementation)	→	Have educational information provided for patients to interpret results (e.g., normal lab ranges)
Recognize the digital space as an opportunity to understand and address user needs through open discussions and collaboration (Patient Engagement, Shared-Decision Making)	*Advocate* for co-design practices where patients and their families are involved. (Dig Readiness, Tech Design)	→	Be co-designed with features that create a sense of connection and build trust between any end-users (e.g., How can connection be maintained for populations that are visually/cognitively impaired, have mental health concerns or underhoused?)
Identify opportunities where digital health technologies can be used to alleviate suffering and improve patient-oriented outcomes (Dig Readiness, Tech Design)	*Create* treatment plans in partnership with patients that enable patients to have choice based on their preferences and comfort level with digital tools (Patient Engagement, Shared-Decision Making)		Ensure the user experience includes user-friendly interfaces and layouts, making its use intuitive and accessible for those who face barriers to use.

Appendix 5: List of Digital Compassion Professional Competency and Technology Attribute Statements Grouped from the eDelphi study

PROFESSIONAL COMPETENCIES			TECHNOLOGY ATTRIBUTES
**Provider should…**			**Technology should…**
**Before the clinical encounter**	**During the clinical encounter**	**After the clinical encounter**	
Identify how compassionate care delivery is perceived by end-users (both patients and providers) during the design and development of a digital tool by taking a human-centered design approach. (Dig Readiness, Tech Design)	→	→	Have an option to give access to or facilitate collaboration with other health care professionals in a patient's circle of care.
Identify education opportunities for major stakeholders (patients, families) to improve digital literacy and digital communication methods. (Dig Readiness, Tech Implementation)	→	→	Create a simple and clear process to give access to or facilitate engagement with family or informal caregivers so that they can be involved to the extent determined by the patient or their directives.

Appendix 5: List of Digital Compassion Professional Competency and Technology Attribute Statements Grouped from the eDelphi study

PROFESSIONAL COMPETENCIES			TECHNOLOGY ATTRIBUTES
**Provider should…**			**Technology should…**
**Before the clinical encounter**	**During the clinical encounter**	**After the clinical encounter**	
**PATIENT PREFERENCES**			
***Prepare* colleagues and the practice environment so that patients are able to use their preferred communication method and digital health technology. (Patient Engagement, Shared-Decision Making)**	***Assess* when and which digital health tools to employ, and implement the tools to allow for patient and family preference. (Dig Readiness, Tech Implementation)**	***Implement* patient preferences for follow-up forms of communication and appointments, (I.e., synchronous, asynchronous or in-person). (Patient Engagement, Shared-Decision Making)**	
Recognize personal biases and assumptions regarding factors that can influence health outcomes (e.g., education, culture, economic and housing stability, digital health literacy) of your patients and their families. (Relationship Building, Professionalism)<br><br>*Recognize* the value of in-person clinical experiences with patients and families and how they align and integrate with digital health tools, patient preference and quality care. (Patient Engagement, Shared-Decision Making)<br><br>*Acknowledge* varying patient experiences with using technology (synchronous or asynchronous communication) and to the extent possible to create time for patients to share their experiences (Patient Experience, Patient Engagement)	*Discuss* patient access, ability and preference for the use of digital health technologies for continuity of care. (Patient Engagement, Patient Experience)<br><br>*Demonstrate* adaptability around technologies to meet patients' needs in the moment (Dig Readiness, Tech Implementation)		Provides personalized prompts, feedback or education materials and considers users' functional, cognitive and emotional needs. (e.g., push notifications to facilitate proactive follow-up or messaging)

Appendix 5: List of Digital Compassion Professional Competency and Technology Attribute Statements Grouped from the eDelphi study

PROFESSIONAL COMPETENCIES			TECHNOLOGY ATTRIBUTES
**Provider should…**			**Technology should…**
**Before the clinical encounter**	**During the clinical encounter**	**After the clinical encounter**	
Establish communication response times for email & text-based messaging, so all stakeholders can expect when messages are addressed and responded to. (Relationship Building, Continuity of Care)	*Plan* follow-up communication with patients who will be receiving lab or imaging results through digital health tools, such as patient portals. (Relationship Building, Continuity of Care)	*Assess* the follow-up communication plan and whether patients felt like they could ask their provider questions to understand their results and any required actions or follow up.	

Appendix 5: List of Digital Compassion Professional Competency and Technology Attribute Statements Grouped from the eDelphi study

**PROFESSIONAL COMPETENCIES**			**TECHNOLOGY ATTRIBUTES**
**Provider should…**			**Technology should…**
**Before the clinical encounter**	**During the clinical encounter**	**After the clinical encounter**	
**THERAPEUTIC RELATIONSHIP**			
Review **and use patient generated data in addition to patient history prior to consultation (Relationship Building, Professionalism)**	***Demonstrate*** **compassion and respect when communicating with patients and families through a digital medium**	***Self-assess*** **digital therapeutic relationship with patients and families. (Relationship Building, Trust)**	
Develop strategies and opportunities to integrate patient generated data into health assessments, patient history notes, and/or care planning, to the extent possible. (Patient Engagement, Patient Experience)	Be mindful of how often and for how long eye contact is broken to look at technology during a clinical encounter (e.g., an electronic record, computer, phone, second monitor, etc) (Relationship Building, Professionalism)<br><br>Use plain language, maintaining eye contact, and being aware of body language and emotional expression (Relationship Building, Professionalism)<br><br>*Apply* verbal/visual cues to acknowledge the suffering and range of emotions seemingly experienced by patients and families when using a digital medium. (Relationship Building, Trust)<br>*Apply* active listening and sensitivity and respond to patients and families in a thoughtful and personalized	*Reflect* on resulting behaviours when digital fatigue occurs and strategies for de-compression (e.g., taking a break from technology). (Relationship Building, Professionalism)<br><br>*Identify* elements that detract from care in a digital format, and devise solutions for improving experiences for patients and providers. (Dig Readiness, Tech Evaluation)<br><br>*Evaluate* how digital health tools function to enable users (both patients and providers) to express empathy and emotion with one another. (Dig Readiness, Tech Design)	

PROFESSIONAL COMPETENCIES			TECHNOLOGY ATTRIBUTES
**Provider should…**			**Technology should…**
**Before the clinical encounter**	**During the clinical encounter**	**After the clinical encounter**	
	way. (Relationship Building, Professionalism)<br><br>*Demonstrate* relatability and honesty by using humility and sharing personal experiences and difficulties using the technology. (Relationship Building, Trust)<br><br>*Deliver* challenging, complete and difficult news to patients and families in their preferred form of communication (e.g., telephone, video, email, direct messaging or in person)	*Ensure* mechanism is in place for patient's feedback on technology use to be collected and addressed accordingly (e.g., directed to the attention of IT support)	

Appendix 5: List of Digital Compassion Professional Competency and Technology Attribute Statements Grouped from the eDelphi study

PROFESSIONAL COMPETENCIES			TECHNOLOGY ATTRIBUTES
**Provider should…**			**Technology should…**
**Before the clinical encounter**	**During the clinical encounter**	**After the clinical encounter**	
**PATIENT SAFETY**			
Develop **risk assessment plans and strategies available so that everyone knows what to do if a crisis should arise (e.g., patient crisis).**	***Assess*** **the safety of patient's physical and emotional environment during telephone and virtual consultations and have a crisis plan if required. (Relationship Building, Trust)**		
Plan phrases or signals with patients to communicate if a patient's environment is no longer safe or private to engage in a discussion about their health. (Relationship Building, Trust)	→		Have a notification feature for when if the space or connection is no longer safe or secure.<br><br>Have communication features for patients to send urgent support requests (during moments of high anxiety or concern) in a safe and secure ‘Live Chat’.

**PROFESSIONAL COMPETENCIES**			**TECHNOLOGY ATTRIBUTES**
**Provider should…**			**Technology should…**
**Before the clinical encounter**	**During the clinical encounter**	**After the clinical encounter**	
**TECHNOLOGY SAFETY**			
***Devise* strategies to manage potential conflict with your patient(s) for when technology fails**	***Confirm* the security and confidentiality of the digital applications and equipment that are being used (e.g., secure Internet connection, personal vs. public computer) and how data is stored. (Relationship Building, Trust)**		Have a notification feature for users (patients and providers) when connectivity or system failures occur.
Understand the privacy implications, potential risks, and how communications and/or data will be secured.	*Notify* and follow up with patients when a technology (synchronous or asynchronous communication) malfunctions or fails. (Dig Readiness, Tech Implementation)	→	Have clear instructions on who to contact and how to contact them if connectivity or system failures occur.<br><br>Enable users (patients and providers) to run a diagnostic of the system to verify the reliability and confidentiality (e.g., stability of Internet connection).
